# Age-related differences in the legibility of degraded text

**DOI:** 10.1186/s41235-016-0023-6

**Published:** 2016-12-12

**Authors:** Benjamin Wolfe, Jonathan Dobres, Anna Kosovicheva, Ruth Rosenholtz, Bryan Reimer

**Affiliations:** 1 AgeLab, Massachusetts Institute of Technology, 77 Massachusetts Ave, E40-278, Cambridge, MA 02139 USA; 2 Department of Psychology, Northeastern University, 360 Huntington Ave, Boston, MA 02115 USA; 3 Department of Brain and Cognitive Sciences, Massachusetts Institute of Technology, 77 Massachusetts Ave, 32-D532, Cambridge, MA 02139 USA

**Keywords:** Noise Condition, Lexical Decision Task, Psychometric Function, Young Participant, High Spatial Frequency

## Abstract

Aging-related changes in the visual system diminish the capacity to perceive the world with the ease and fidelity younger adults are accustomed to. Among many consequences of this, older adults find that text that they could once read easily proves difficult to read, even with sufficient acuity correction. Building on previous work examining visual factors in legibility, we examine potential causes for these age-related effects in the absence of other ocular pathology. We asked participants to discriminate words from non-words in a lexical decision task. The stimuli participants viewed were either blurred or presented in a noise field to simulate, respectively, decreased sensitivity to fine detail (loss of acuity) and detuning of visually selective neurons. We then use the differences in performance between older and younger participants to suggest how older participants’ performance could be approximated to facilitate maximally usable designs.

## Significance

Age-related changes in visual perception have been extensively studied in clinical and laboratory settings, but are seldom considered in applied contexts from a psychophysical point of view. A user’s perception of the world will change as they age, but how does that impact how easily they can read text at a glance? Reading a newspaper at home is a fundamentally different form of reading than glancing at a smartphone or trying to figure out which button is which in an in-vehicle display. This work simulates two of the major components of age-related changes in visual perception with older and younger observers. Accounting for these differences when designing interfaces will improve usability and the user experience for older and younger users alike.

## Background

How easy is it for you to read this sentence? Its legibility may depend on the typeface the journal uses, the font size, the page’s background color, and the room lighting, to say nothing of how close or far away the screen is. These are all physical features of the article itself and how the journal has chosen to typeset it, rather than features of your visual system. Much of the long history of legibility research has investigated visual factors inherent to the text itself (beginning with Paterson & Tinker, 1932; Sanford, 1888, among many others), rather than the limitations of the visual system. How physical factors impact legibility is far from a new question; previous work in this domain has examined the impact of the shape and form (Paterson & Tinker, 1932; Roethlein, 1912) of the typeface itself, the size of the letterforms (Sanford, 1888), the polarity of the display (Piepenbrock, Mayr, & Buchner, 2014; Piepenbrock, Mayr, Mund, & Buchner, 2013) among other features. Rather less research has examined the combined problem of modern digital typefaces and the aging visual system (although, see Rayner, Reichle, Stroud, Williams, and Pollatsek (2006) for an overall treatment of the question of age and reading; and, for that matter, the relatively recent shift to reading on displays (versus paper) is also a new domain (c.f. Dillon, 1992).

Beyond the transition to screens rather than printed surfaces, there are now many settings where we glance at a screen and try to read a single word. Previous research has suggested (Uchida, Kepecs, & Mainen, 2006) that studies of longform reading, while certainly related to reading at a glance, may not adequately explain the particular challenges inherent in glance reading. Of particular interest to us, in longform reading, there is time for the reader to begin to puzzle out the letterforms and words presented, but reading a single word at a glance does not offer the same opportunities. In particular, glance legibility is a particular concern with smart devices—a user might glance at their smartphone or smartwatch and read a single word, or, while driving, may glance to their navigation display to learn the name of the street they have to look for. In these settings, the user will never read more than a word or two—and, perforce, they must do so exceedingly quickly.

### Glance legibility in real-world settings

Recent work on glance legibility (Dobres, Chahine, Reimer, Gould, & Mehler, 2016) and word recognition (Balota, Yap, & Cortese, 2006) has shown that legibility in the context of word recognition is impacted by both the typeface used (Sheedy, Subbaram, Zimmerman, & Hayes, 2005), character size (Nazir, Jacobs, & O’Regan, 1998), and display polarity, as well as the age of the reader. For that matter, earlier work on glance legibility has examined similar questions in the context of road signage (Forbes, 1939) and letter case for single words (Arditi & Cho, 2005; Balota et al., 2006; Breland & Breland, 1944; Nazir et al., 1998). Building on these findings, we note that work to date on glance legibility has merely demonstrated that performance decreases with age; our goal is to examine some of the changes that occur in the aging visual system and determine how they may impact legibility for older and younger users using psychophysical methods. Moreover, being able to quantify and visualize the perceptual consequences of these changes could be profoundly useful in an applied context by facilitating both understanding of age-related changes in legibility and development of maximally usable designs. Knowing that older readers find text presented in certain ways harder to read (Aberson & Bouwhuis, 1997; Mitzner & Rogers, 2003) is useful, but being able to quantify and visualize the impact of particular age-related factors on perception is vastly more helpful. By allowing a designer to experience how older adults will experience their design, we can enable them to better understand their user’s future experience, facilitating design that can be used by a range of users, which may reduce the need for costly user studies.

This is a particular concern in driving, with the shift from buttons and gauges to flat screens, because the only reading a driver should be doing while on the road is glance reading—a vehicle that expects the driver to read a paragraph while driving is far from a safe design. Studies of driver behavior have noted profound perceptual changes and associated behavioral consequences in older drivers (Owsley, 2011; Sekuler, Hutman, & Owsley, 1980). However, the intersection of legibility and age is an understudied domain in the context of driving, particularly in light of the increasing use of digital displays in the car, in lieu of the combination of small displays and physical buttons that dominated until the last decade. With this recent change, it is no longer enough to simply memorize the physical layout of buttons in the cabin; the driver must be able to read the button before acting, which, in turn, requires them to keep their eyes off the road for a longer period of time, which increases the risk of a collision.

However, in-vehicle displays are far from the only context in which text is read at a glance by users of all ages. Perhaps an even more immediate example is the recent mass adoption of smartphones and the progressive adoption of smartwatches and other wearable computing devices. In fact, 2.4 billion smartphones were sold worldwide through 2014 (GSMA, 2015), with a nearly 90% adoption rate by users in the US aged 13–34 years, and a 50% adoption by users older than 65 years, as of March 2015 (Lipsman, 2015). Investigating the ways in which glance legibility changes for people of different ages is key for making these devices, as well as displays in the car, usable by the largest number of users.

### Aging-related changes in visual perception

The perceptual requirements of a user in their 20s are vastly different than those of a user in their 60s. The sensitivity and capabilities of the human visual system change with age, with peak functionality around the age of 25 years, and slowly diminsh thereafter (Owsley, 2011; Owsley, Sekuler, & Siemsen, 1983). These changes include: a reduction in sensitivity to high spatial frequencies (small details), resulting in perceived blur; shifts in the flexibility of the lens; changes in the shape of the eye; as well as changes in the underlying behavior of cells in the visual cortex, all of which will degrade a person’s perception of the world. These changes merely reflect the normal aging process and its attendant effects on visual perception. Beyond normal aging, there are a wide array of pathologies (e.g. macular degeneration, retinitis pigmentosa, and cataracts) that can further diminish or entirely eliminate a person’s ability to see the world. While there is no clear line dividing the effects of aging from those of pathology—particularly in the case of cataracts, which are inevitable in the aging eye—we focus on two universal changes that occur in the aging visual system: first, the diminished sensitivity to high spatial frequency information (Owsley et al., 1983), and second, the increase in perceptual noise at the neuronal level (Schmolesky, Wang, Pu, & Leventhal, 2000).

The reduction in sensitivity to high spatial frequencies is comparatively simple to consider; with aging, the detail that can be seen is reduced, with this reduction disproportionately affecting the high spatial frequencies responsible for fine spatial detail (Owsley, 2011). In essence, the ability to see fine details is slowly lost, although this has only limited impact on many visual tasks (Pardhan, 2004). In fact, some evidence suggests that older participants are better able to cope with a greater degree of optical blur than younger participants (Kline, Buck, Sell, & Bolan, 1999).

The increase in perceptual noise from broadened tuning of visually selective neurons, however, is less intuitive. Detuned neurons fire less discriminately, resulting in a less accurate representation of the stimulus. A neuron that might have fired strongly to orientations within 10° of its preferred orientation might, for example, fire strongly to orientations within 15° or 20° of its preferred orientation. Notably, this detuning not due to changes in the optics of the eye or in the retina, but rather to a change in how the brain represents the signals received from the retina. This increase in internal noise has been most extensively studied in animal models of aging, particularly the single-unit recording work of Schmolesky et al. (2000), showing decreased specificity of tuning for neurons in early visual cortex in older rhesus monkeys. Similar results have been shown in other animal models (e.g. rats (Mendelson & Wells, 2002) and cats (Betts, Sekuler, & Bennett, 2007)) and analogous results have been found in human behavioral research (Grady et al., 1994; Johnson, Adams, & Lewis, 1989). Work with human participants has shown that a decrease in sensitivity to high spatial frequencies (blurring them beyond recognition) and an increase in internal neuronal noise may interact in deleterious ways for older participants (Bennett, Sekuler, & Ozin, 1999), suggesting that examining both these factors in the context of glance legibility may yield insights of particular relevance for the applied settings we have mentioned.

One can mimic detuning effects by adding external noise to stimuli, although this is an imperfect approximation. External noise added to an oriented bar, for instance, will theoretically reduce firing of a neuron tuned to that bar’s orientation, while increasing the probability that neurons tuned to other orientations will respond. While adding external noise is unlikely to mimic the perceptual experience of an older participant, it allows us to behaviorally explore the effects of increased internal noise. Along these lines, some conceptually similar work, on the question of how decreased contrast impacts reading performance, has been performed with groups of older and younger participants to better understand how they differ (Mitzner & Rogers, 2003). In the case of our study, adding external noise will effectively broaden neuronal tuning relative to the undegraded stimulus, increasing noise in participants’ responses.

To understand the impact of this subset of age-related effects on vision and, more specifically, on glance legibility, we performed two experiments in which participants were asked to perform a lexical decision task on degraded stimuli. To simulate the effects of diminished sensitivity to high spatial frequencies, we blurred our lexical stimuli to various degrees; at a modest level of blur, the words are blurry but recognizable, and at a greater degree, they become entirely unrecognizable. To simulate decreases in neuronal specificity, we presented stimuli integrated into fields of 1/f noise at a range of contrasts, up to the point where the lexical stimulus was utterly indistinguishable from the background.

We recruited two groups of participants, one in their 20s and one in their 60s, to compare performance between them in order to ask how our manipulations changed legibility at different ages. In our first experiment, ambient illumination was kept low, to focus on the degradation of the stimuli; in our second experiment, we added a condition with high amounts of diffuse ambient illumination, simulating viewing a digital display on an overcast day (e.g. using a smartphone outdoors or driving a car with modern digital displays in the cabin during the day). Overall, our results help quantify how legibility changes as a function of age, and, critically, how we might simulate how an older user might see and experience an interface.

## Experiment 1: legibility of degraded text under low ambient illumination

### Materials and methods

#### Participants

A total of 37 participants were recruited for the experiment, five of which were excluded from the final analysis. One participant was withdrawn due to low acuity, three participants were withdrawn for mean reaction times in excess of 1000 ms, and one participant was excluded because we had achieved the needed gender and age distribution. All other participants had normal or corrected to normal acuity, as assessed using both the Federal Aviation Administration’s test for near acuity (Form 8500-1), and the Snellen Eye Chart for distance acuity. All data reported were from a final set of 32 participants (16 men). The sample was additionally divided into older and younger cohorts (16 participants in each; 8 men, 8 women), with the younger participants in the age range of 20–29 years (mean age, 24.1 years) and the older participants in the age range of 60–69 years (mean age, 64.4 years). All participants provided informed consent prior to data collection in accordance with the requirements of MIT’s Committee on the Use of Humans as Experimental Subjects (COUHES) and the Declaration of Helsinki.

#### Apparatus, stimuli, and procedure

*Apparatus*. All stimuli were presented using PsychoPy (Peirce, 2007, 2008) on a Mac Mini (Apple Computer, Cupertino, CA, USA). Stimuli were displayed on a 68 cm Acer LCD display (Model B276HI) at a resolution of 1920 × 1200 pixels with a refresh rate of 60 Hz and a viewing distance of 70 cm. Head position was unconstrained, allowing for a degree of positional variability likely to be encountered in real-world viewing scenarios. Participants performed the task in a dimly lit (~10 lux) room.

*Stimuli*. All stimuli were six-letter words or non-words, as used by Dobres et al. (2016) with the words originally selected from the MCWord database of unique wordforms by Medler and Binder (2005). Stimuli in the experiment were generated in the humanist sans serif typeface Frutiger, for comparability with previous work by the co-authors (c.f. Dobres et al., 2016; Dobres, Chahine, Reimer, Mehler, & Coughlin, 2014), and rendered at 4 mm (0.33°) capital letter height onscreen. While we use capital letter height as the measure of optical size, in accordance with previous work in this area, all stimuli consisted of lowercase letters. Non-degraded stimuli consisted of white text (223 cd/m^2^) on a black (0.34 cd/m^2^) background (negative polarity), measured at the display surface with a Gossen Mavo-Monitor luminance meter. Negative polarity was used to maximize observed differences between conditions, based on previous work with this typeface by the authors. Negative polarity is commonly used for in-vehicle displays under low ambient illumination conditions.

**Fig. 1:**
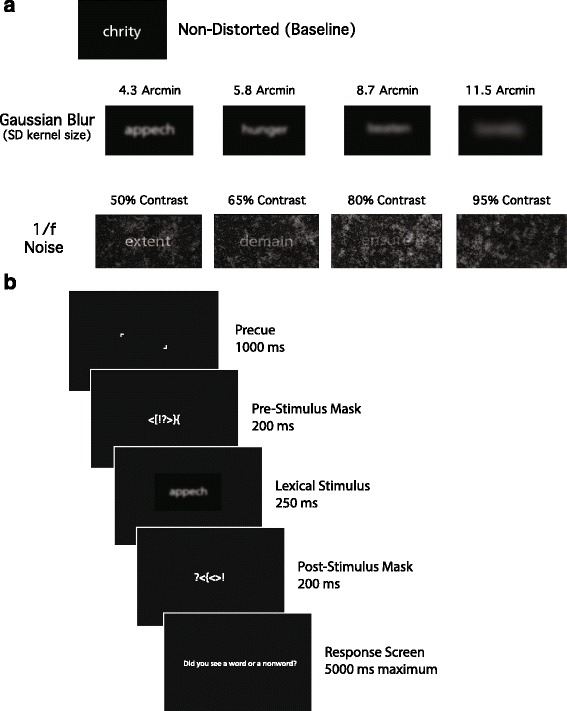
**a** Illustration of all stimulus levels in blur and noise conditions for Experiments 1 and 2, text generated in Frutiger. **b** Stimulus sequence for a trial in both experiments

To assess the differential impacts of blur and noise, respectively, on legibility for older and younger participants, we used two independent degradation conditions in our experiments. To simulate the reduced sensitivity to high spatial frequencies, on some trials we blurred our stimuli. On other trials, to approximate the effects of broadening of neuronal tuning, we presented our lexical stimuli in a field of noise (see Fig. 1) to diminish their discriminability (Damera-Venkata, Kite, Geisler, Evans, & Bovik, 2000; Michel, Chen, Geisler, & Seidemann, 2013). While these degradations are imperfect representations of the effects of aging, these transformations allow us to examine specific facets of age and legibility. We note that the gradual nature of aging means that our older participants may have developed compensatory strategies for similar changes in their visual systems; however, the synthetic nature of our degradations should reduce the effectiveness of any compensatory strategies.

In the trials where we blurred the stimuli, this was accomplished by convolving full contrast text images with a Gaussian kernel of different sizes to achieve different levels of blur. The standard deviations of the Gaussian blur kernels used in this experiment were 4.3, 5.8, 8.7, and 11.5 arcmin (for our 70 cm viewing distance), based on pilot testing. Increasing the standard deviation increases the image blur and decreases the available resolution. In our noise trials, we added a field of 1/f noise to the text image (Fig. 1a) at different levels of noise contrast. Noise contrast levels were chosen based on pilot testing to assess a full range of performance, from ceiling to chance. Noise patches were 2.4° high and 4.8° wide and had one of four contrast levels: 50, 65, 80, and 95%. The contrast of the full image (noise with text) was maintained at 100% for each noise contrast condition. Both the blur and noise conditions also included a no-degradation condition (0 arcmin of blur, 0% noise contrast) as a baseline for a total of five levels in each condition.

*Procedure*. Each trial consisted of the following sequence (Fig. 1b). First, a precue was presented for 1000 ms at the center of the screen to indicate the region where the lexical stimulus would be presented. The precue consisted of four “L” shapes (0.48° on a side) rotated and positioned to form the corners of a rectangle subtending 4.8° horizontally and 2.4° vertically. No stimuli were presented outside the region indicated by the rectangular cue. This was followed by a 200 ms screen-centered mask consisting of a string of eight random punctuation characters (selected with replacement from: =, ^, <, >, and |). Following this mask, participants were shown a set of letters that had an equal probability of forming a word or a non-word. Six-letter words and non-words were selected randomly without replacement from separate lists of 299 and 291 alternatives, respectively, and had a randomly selected level of either blur or noise. All word and non-word stimuli were presented for 250 ms, immediately followed by a different random punctuation mask, presented for 200 ms.

Following the final mask, participants were instructed to respond as to whether the lexical stimulus was a word or a non-word by pressing a key on the keyboard. They were given a warning display if they took longer than 5000 ms to respond. Trials in which reaction times exceeded 5000 ms were excluded from the analysis (0.016% of all trials). There were 20 trials for every unique combination of stimulus category (word versus non-word), degradation type (noise versus blur), and degradation level (five levels) for a total of 400 trials per participant. Trial order was randomized for each participant and the experiment was divided into eight blocks of 50 trials with breaks between each block.

Prior to the start of the experiment, participants performed a small set of practice trials until they had correctly completed five consecutive trials. In these practice trials, the lexical stimuli were presented without any blur or noise, generated in the typeface Georgia, and presented for 1000 ms, rather than the 250 ms in the main experiment. Participants also received visual feedback regarding their accuracy on each trial during the practice phase of the experiment. No feedback regarding accuracy was provided during the main experiment.

*Analysis*. For each participant and type of degradation (blur and noise), we used maximum likelihood estimation to fit a two-parameter psychometric function, a cumulative Normal to the lexical decision accuracy as a function of degradation level:

1 $$ \Phi (x)=\frac{1}{2}+\frac{1}{2\sigma \sqrt{2\pi }}{\displaystyle \underset{-\infty }{\overset{x}{\int }}}{e}^{-\frac{{\left(t-\mu \right)}^2}{2{\sigma}^2}}dt $$

where μ represents the mean (horizontal shift) and σ represents the standard deviation (slope). Mean goodness of fit for the blur condition, averaged across participants was, R^2^ = 0.93; for the noise condition, mean R^2^ = 0.81. The critical question is how the performance curves differ for older versus younger participants. To this end, differences between age cohorts were tested with two-tailed unpaired Welch’s t-tests and effect size was determined using Cohen’s *d*.

In addition to this fit-based analysis, we also performed an accuracy-based analysis for both the blur and noise conditions, in which we compared percent correct performance between the two age groups, at each level of degradation, as an additional verification of our findings in the fit-based analysis. We performed two separate mixed-model ANOVAs, one for each degradation type (noise and blur), with age group as a between-subjects factor and the five degradation levels (either noise contrast or blur in arcminutes) as a within-subjects factor. Effect sizes are reported as eta-squared.

Finally, each comparison includes an estimate of the corresponding Bayes factor of the alternative hypothesis (H_1_) against the null (H_0_), reported as *BF*_*10*_, and calculated using the Jeffrey-Zellner-Siow prior (Zellner & Siow, 1980). Values of *BF*_*10*_ that are greater than 1 indicate that the observed data are more likely under the alternative than the null. The converse is true for values of *BF*_*10*_ that are less than 1 (i.e. the observed result is more likely under the null).

### Results

#### Analysis of psychometric functions for older versus younger adults

While both types of degraded trials were interleaved in our experiment, we will discuss them separately for clarity, as they are two entirely independent stimulus manipulations.

**Fig. 2:**
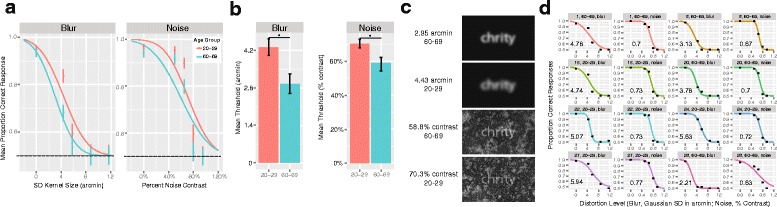
Experiment 1, group and individual performance in blur and noise conditions. **a** Mean performance, split by age group, fit to cumulative Gaussian functions. *Dotted horizontal lines* represent chance (50%) performance on the task. **b** *Bar plots* show mean 75% performance thresholds by age group. In both the blur and noise conditions, these thresholds are significantly different at the group level, *p* < 0.001. *Error bars* are 95% bootstrapped confidence intervals. **c** Stimuli with blur and noise at the 75% mean threshold level shown in (**b**), separated by age group. **d** Exemplar individual participant data from eight participants (four per age group), for blur and noise conditions, fit to cumulative Gaussian functions. Values *inset* in lower left of each subplot are the 75% performance thresholds for each participant and each condition

In the blur condition, we find a significant shift in the psychometric function between older and younger observers (*t*(28.9) = 3.57, *p* = 0.001, *d* = 1.26, *BF*_*10*_ = 25.89). Specifically, it is useful to consider the midpoint of the psychometric function, the 75% correct threshold. Compared to younger observers, accuracy for older observers dropped to 75% correct at a lower level of blur (2.95 versus 4.43 arcmin; Fig. 2a; see Fig. 2b for threshold by age group and Fig. 2d for exemplar individual participant data). Similarly, in the noise condition, older observers had lower 75% thresholds (i.e. worse performance) than younger observers (*t*(27.2) = 3.70, *p* < 0.001, *d* = 1.31, *BF*_*10*_ = 34.34). Accuracy for older observers dropped to 75% at a lower noise contrast level than it did for younger observers (58.8 versus 70.3% contrast). Therefore, in order to equate performance between younger and older participants in the blur condition, the Gaussian kernel SD would need to be increased by 1.48 arcmin for the stimuli presented to the 20–29 age group. To do the same in the noise condition, an additional 11.5% noise contrast would need to be added. The group thresholds are visualized in Fig. 2c.

To determine whether there was any difference in how steeply performance declines (as blur or noise increases), we compared the fitted slope parameters (σ) between the age groups. There was no difference between the 20–29 age group and the 60–69 age group for either the blur (2.72 versus 2.28, *t*(29.9) = 1.11, *p* = 0.28, *d* = 0.39, *BF*_*10*_ = 0.54) or the noise conditions (0.25 versus 0.34, *t*(27.9) = −1.50, *p* = 0.15, *d* = 0.53, *BF*_*10*_ = 0.78). Therefore, any differences between the age groups are best summarized as a lateral shift in the psychometric function, without a difference in slope.

As we will discuss later, knowing the shift of the psychometric function with age is particularly useful for providing design intuitions, because it provides a single value that describes the differences between older and younger participants. One can, of course, also look at other points on the curve, if that is of relevance for a particular research question, e.g. how would we expect older adults to respond to a slightly blurred user interface compared to younger adults. If the psychometric functions were perfect cumulative Normal functions with no change in slope nor asymptotic performance, we would observe the same shift in 90% thresholds as we observe in 75% thresholds, but of course none of these assumptions holds exactly. In the blur condition, we observed a trending difference in the 90% threshold between the two age groups, with older observers’ performance dropping to 90% at a lower level of blur compared to younger observers (1.04 versus 2.16 armin, *t*(29.28) = 2.0, *p* = 0.06, *d* = 0.71, *BF*_*10*_ = 1.47). In the noise condition, the difference between the two age groups was significant, *t*(27.05) = 2.48, *p* = 0.02, *d* = 0.88, *BF*_*10*_ = 3.13. Compared to younger observers, older observers required a lower level of noise in order for performance to drop to 90% (30 versus 49% contrast).

#### Accuracy analysis

While the differences between the psychometric functions detailed in the previous section are highly informative, it is also valuable to verify those results using a complementary method. In the blur condition, an ANOVA on percent correct responses showed a significant main effect of age group (*F*(1,30) = 12.13, *p* = 0.002, *η* ^*2*^ = 0.29, *BF*_*10*_ = 10.63), with lower accuracy in the 60–69 age group than in the 20–29 age group (63.0 versus 67.8%, respectively). As expected, there was a significant main effect of blur level (*F*(4,120) = 307.31, *p* < 0.001, *η* ^*2*^ = 0.89, *BF*_*10*_ = 4.10 × 10^62^), with performance decreasing as the level of blur increased. This result would be expected regardless of any differences between the two age groups and indicates that our blur manipulation reduced accuracy in the lexical decision task. The interaction between age group and blur level was also significant, *F*(4,120) = 9.02, *p* < 0.001, *η* ^*2*^ = 0.03, *BF*_*10*_ = 1.34 × 10^4^. Comparisons between the two age groups at each level of blur (using a Šidák-corrected alpha of 0.01) showed a significant effect at 4.3 arcminutes (*t*(30) = 4.14, *p* < 0.001, *d* = 1.47, *BF*_*10*_ = 95.48). The difference between the two age groups was not significant at any of the remaining blur levels, including the no-blur condition (all *p* values > 0.08, *BF*_*10*_ < 1.15).

An ANOVA on participants’ performance in the noise condition yielded similar results. There was a significant main effect of age group (*F*(1,30) = 19.12, *p* < 0.001, *η* ^*2*^ = 0.39, *BF*_*10*_ = 85.19), with lower overall accuracy in the 60–69 age group than in the 20–29 age group (73.4 versus 78.4%). The effect of noise level was also significant (*F*(4,120) = 532.07, *p* < 0.001, *η* ^*2*^ = 0.94, *BF*_*10*_ = 1.49 × 10^82^), indicating that the noise manipulation reduced participants’ performance. Finally, we observed a significant interaction between age group and noise level, *F*(4,120) = 4.55, *p* = 0.002, *η* ^*2*^ = 0.008, *BF*_*10*_ = 23.43. The difference between the 20–29 age group and the 60–69 age groups was significant at both the 65% (*t*(30) = 3.98, *d* = 1.41 *p* < 0.001, *BF*_*10*_ = 64.61) and the 80% noise contrast levels (*t*(30) = 3.53, *d* = 1.24, *p* = 0.001, *BF*_*10*_ = 23.98). The difference between the age groups was not significant at the remaining contrast levels (all *p* values > 0.09, *BF*_*10*_ < 1.07), including the no-contrast level.

Together, the results from the accuracy analysis are consistent with the psychometric fitting results. In both the blur and the noise conditions, we see a significant difference between the two age groups only at intermediate levels of blur (or noise) and not at the extremes (i.e. the lowest and highest levels of blur or noise). This pattern of results is consistent with a lateral (horizontal) shift of a sigmoid function, which produces larger differences in the y-values (percentage correct) at intermediate x-values (e.g. intermediate levels of blur) and a smaller difference at the extremes.

#### Reaction time

Finally, we analyzed observers’ mean reaction times using a separate 5 (degradation level) × 2 (age group) mixed-model ANOVA for each degradation type. In the blur condition, there was a significant main effect of age group (*F*(1,30) = 15.51, *p* < 0.001, *η* ^*2*^ = 0.34, *BF*_*10*_ = 47.97), with older participants responding more slowly than younger observers (620.8 ms and 399.6 ms, respectively). Neither the main effect of blur level (*F*(4,120) = 0.81, *p* = 0.52, *η* ^*2*^ = 0.03, *BF*_*10*_ = 0.06) nor the interaction between blur level and age group reached significance (*F*(4,120) = 0.89, *p* = 0.47, *η* ^*2*^ = 0.03, *BF*_*10*_ = 0.15).

In the noise condition, the main effect of age group was also significant (*F*(1,30) = 18.94, *p* < 0.001, *η* ^*2*^ = 0.39, *BF*_*10*_ = 137.61), with slower mean reaction times in the 60–69 age group (619.7 ms) than the 20–29 age group (392.0 ms). Unlike the blur condition, there was a significant main effect of degradation level, *F*(4,120) = 3.64, *p* = 0.008, *η* ^*2*^ = 0.11, *BF*_*10*_ = 4.36. A trend analysis showed a significant linear trend, indicating that reaction times increased with increasing noise contrast (*F*(1,30) = 5.23, *p* = 0.029, *η* ^*2*^ = 0.15) and pairwise comparisons (with a Šidák-corrected alpha of 0.005) showed significantly slower reaction times in the 65% noise condition (517.6 ms) compared to the 50% noise condition (472.0 ms), *t*(31) = −3.29, *p* = 0.003, *BF*_*10*_ = 14.46. All other pairwise comparisons did not reach significance (*p* > 0.01). Finally, the age group × noise level interaction was not significant (*F*(4,120) = 0.79, *p* = 0.53, *η* ^*2*^ = 0.02, *BF*_*10*_ = 0.13).

Together, these results point to fast lexical decision judgments (with a mean reaction time across age groups of 508.0 ms), with older adults responding more slowly than younger adults in both conditions by more than 200 ms on average. In addition, we observe longer reaction times with increasing noise contrast, indicating that, at least in some cases, reaction times were modulated by task difficulty.

### Discussion

Two findings stand out from this experiment. First, that in the absence of degradation, older and younger participants are both capable of performing our lexical decision task at a high level of accuracy, even if older participants are slower to do so. Second, and much more interestingly, that degraded stimuli, both blurred and with added noise, have a greater detrimental effect on legibility for older participants than younger participants, and that this change can be best and most simply described as a horizontal shift of the function used to fit the data. The fact of this horizontal shift means that it is entirely possible, based on data collected under the low ambient illumination conditions used in this experiment, to simulate the difficulty an older observer has performing the task with a given stimulus and give a younger observer an intuitive appreciation of the differences in their respective perceptions.

## Experiment 2: legibility of degraded text under low and high ambient illumination

While the results of our first experiment suggest that simulating the perceptual experience of older users is possible, stimuli in Experiment 1 were only presented in a dim environment, leading to somewhat ideal conditions for viewing self-illuminated stimuli such as on a computer monitor or smartphone. In the real world, displays are used under a wide array of ambient illumination conditions, many of which reduce visibility considerably. We repeated the experiment with new participants under both low and high ambient illumination conditions. We assess the legibility of degraded stimuli under conditions similar to an overcast day (5000 lux, as compared to ~10 lux in Experiment 1).

### Materials and methods

#### Participants

A total of 40 new participants were recruited for this experiment, eight of whom (7 older, 1 younger) were excluded from the final analysis for failing to achieve a minimum level of performance (75% or greater) overall in either the blur, noise, or non-degraded conditions. As in Experiment 1, all participants had normal or corrected-to-normal acuity, assessed with the Federal Aviation Administration’s test for near acuity (Form 8500-1) and the Snellen Eye Chart for distance acuity. All data reported are from a final set of 32 participants (16 women), additionally divided into older (mean age, 64.9 years) and younger (mean age, 23 years) cohorts with eight men and eight women in each. All participants provided informed consent as required by MIT’s Committee On the Use of Humans as Experimental Subjects.

#### Stimuli

Stimuli were identical to those used in Experiment 1 with the addition of ambient illumination manipulations as detailed below.

#### Ambient illumination apparatus

Illumination for the low ambient condition (10 lux) was provided by the display itself, as in Experiment 1. In the high ambient condition, two Aputure-brand Light Storm LED panels (model LS-1c) were placed behind and to the side of the participant’s chair provided additional illumination. This provided 5000 lux of diffuse ambient illumination, measured at the display with a luminance meter, which is comparable to outdoor ambient illumination on an overcast day.

#### Experimental procedure

The experimental procedure was identical to that in Experiment 1, with a 25% reduction in the number of trials to enable twice as many condition, for a total of 600 trials each, with breaks every 50 trials. Each participant viewed 15 trials per unique combination of stimulus category (word versus non-word), illumination condition (low versus high), degradation condition (noise or blur), and degradation level (five levels, including 0 arcmins of blur or 0% noise contrast).

#### Analysis

As in Experiment 1, trials with response latencies of over 5000 ms were removed from the analysis (0.01% of all trials). Given that the primary goal in Experiment 2 was to examine the effects of ambient illumination on participants’ performance thresholds, we report the results of a psychophysical threshold-based analysis. As before, performance across the five degradation levels from each individual participant within each degradation type (noise or blur) and lighting condition was fit to a cumulative Normal psychometric function (for blur, mean R^2^ = 0.96; for noise, R^2^ = 0.80).

### Results

**Fig. 3:**
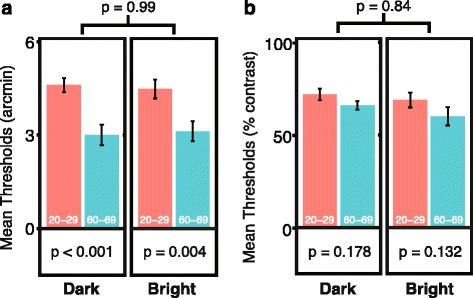
Experiment 2, mean performance thresholds (blur level or noise level at which participants achieved 75% accuracy) for older and younger participants. **a** Mean blur threshold by age group and illumination condition, showing a significant difference by age group but no difference by illumination condition. **b** Mean noise threshold by age group and illumination condition, showing no effect of age group and no difference by illumination condition

Since we were primarily interested in comparing performance between the two age groups and between the two illumination conditions, we performed two separate 2 (age group) × 2 (illumination) mixed-model ANOVAs. Within the blur condition, we observed a significant main effect of age group (*F*(1,30) = 16.61, *p* = 0.0003, *η* ^*2*^ = 0.36, *BF*_*10*_ = 84.96) (Fig. 3a). Compared to older observers, younger observers required an additional 1.50 arcmin of blur (4.60 versus 3.10 arcmin) in order for performance to drop to 75%. However, observers’ thresholds were similar across the bright and dark ambient illumination conditions (*F*(1,30) = 0.0002, *p* = 0.99, *η* ^*2*^ = 7.94 × 10^−6^, *BF*_*10*_ = 0.25) and there was no interaction between illumination and age group (*F*(1,30) = 0.28, *p* = 0.60, *η* ^*2*^ = 0.009, *BF*_*10*_ = 0.37).

Within the noise condition, younger observers required an additional 6.6% noise contrast (70.3 versus 63.7% contrast) in order for performance to drop to 75%. However, in contrast to the results in Experiment 1, the main effect of age group did not reach significance (*F*(1,30) = 2.53, *p* = 0.122, *η* ^*2*^ = 0.078, *BF*_*10*_ = 0.98) (Fig. 3b). We also observed a trending but non-significant effect of ambient illumination (*F*(1,30) = 3.19, *p* = 0.084, *η* ^*2*^ = 0.095, *BF*_*10*_ = 0.96), with higher thresholds (i.e. better performance) under lower ambient illumination compared to the higher ambient illumination (68.9 versus 65.0% contrast). The age group × illumination interaction was not significant (*F*(1,30) = 0.30, *p* = 0.59, *η* ^*2*^ = 0.009, *BF*_*10*_ = 0.38).

As in Experiment 1, we compared the fitted slope parameters between the two age groups and the two lighting conditions (using a 2 × 2 mixed-model ANOVA) to determine whether these factors influence how steeply performance declines (as either blur or noise increases). Within the blur condition, there was no significant effect of age group (*F*(1,30) = 1.98 × 10^−5^, *p* = 0.996, *η* ^*2*^ = 6.59 × 10^−7^, *BF*_*10*_ = 0.38), with similar slope estimates between the two age groups (1.755 versus 1.757 for younger and older participants, respectively). In addition, neither the main effect of ambient illumination (*F*(1,30) = 0.23, *p* = 0.63, *η* ^*2*^ = 0.008, *BF*_*10*_ = 0.30) nor the age group illumination interaction (*F*(1,30) = 0.36, *p* = 0.55, *η* ^*2*^ = 0.012, *BF*_*10*_ = 0.37) were significant.

Similarly, in the noise condition, we observed no significant effect of age group on participants’ slopes (0.24 versus 0.28 for younger and older participants, respectively; *F*(1,30) = 0.78, *p* = 0.39, *η* ^*2*^ = 0.025, *BF*_*10*_ = 0.46). As we observed in the blur condition, neither the main effect of ambient illumination (*F*(1,30) = 0.10, *p* = 0.75, *η* ^*2*^ = 0.003, *BF*_*10*_ = 0.27) nor the age group illumination interaction (*F*(1,30) = 1.66, *p* = 0.21, *η* ^*2*^ = 0.052, *BF*_*10*_ = 0.67) were significant.

Together, these results indicated that the slopes in the blur and noise conditions were similar between the two age groups (as reported in Experiment 1) and were not significantly affected by illumination.

### Discussion

In Experiment 2, we find no significant difference in 75% performance thresholds in either the blur or the noise condition between low and high ambient illumination, demonstrating that ambient illumination is not a significant factor in glance legibility for text presented on screens. Given this lack of an ambient illumination effect, the shift between older and younger participants can be considered constant, regardless of ambient illumination. However, while we find a significant effect of age in the blur condition, in both the low and high ambient illumination, we fail to replicate the effect in the noise condition in both the low and high ambient illumination conditions. While we only partially replicate our results in Experiment 1, we believe that this can be attributed to two differences between our experiments. We found that participants in Experiment 2 failed to achieve the necessary level of performance more frequently than in Experiment 1 (as shown by our increased exclusion rate); this may be attributable to the 50% increase in total trials in Experiment 2. In addition, our failure to replicate our age effect in Experiment 1’s noise condition may be a result of a 25% reduction in the number of trials per condition, as well as increased participant fatigue as a result of the increased experiment duration.

These issues notwithstanding, the core result of Experiment 2 is that, for single words presented on a digital display, ambient illumination does not have a significant effect on performance thresholds. With this in mind, the differences between the group thresholds can be considered essentially robust to ambient illumination, allowing us to suggest one value for blur and noise, respectively. Given that our goal in this work is to provide the framework for a simple visualization of reduced legibility for older adults, this lack of a difference indicates that the same amount of additional blur or noise can be used essentially regardless of ambient illumination.

## General discussion

Glancing at a word and being able to read it quickly and easily is seemingly simple, but as we age, this becomes more difficult, to the point where it may adversely impact users’ ability to use their car or smartphone. To better understand how the aging process changes glance legibility, we simulated two of these degradations in our experiments and thereby gained a better understanding of their consequences for legibility. That there are differences between older and younger participants is not surprising, but the pattern of differences between older and younger participants demonstrates that older participants’ difficulties reading text can be simulated for younger participants in a simple and straightforward manner, by applying the appropriate blur or added noise to a given design. This can be done essentially regardless of ambient illumination, although other factors, such as glare or reduced retinal illuminance, may require further experiments beyond the scope of this paper (c.f. Kline, 1994; Owsley et al., 1983; Sloane, Owsley, & Alvarez, 1988). In our experiments, when we blurred our stimuli, we found a decreased threshold in older participants compared to younger participants, indicating that lower levels of blur had a deleterious effect on older participants’ ability to perform the task. We found a shift in psychometric functions between younger and older participants for the blurred stimuli, indicating that younger participants viewing a stimulus with 1.48 arcminutes more blur (at 70 cm viewing distance) perform similarly to older adults.

These results, at first glance, appear to be at odds with previous work in related domains. Earlier work on road sign legibility showed that older participants’ performance was more robust to blur than that of younger participants (Kline, Buck, Sell, Bolan, & Dewar, 1999). However, attempting to read a continually presented road sign as it increases in size is a substantially different task than attempting to read a briefly presented word, visible only for 250 ms, which could easily account for the differences in our results. The robustness to blur that Kline et al. observed may also be a function of driver experience; older drivers will have seen (and learned to identify) the signs used in the experiment over a longer period of time than the younger participants and will, therefore, have had time to develop compensatory strategies to account for changes in their perception of the world over time.

In contrast to the work of Kline et al., we observed that levels of blur which only barely impacted the performance of younger participants had a profound impact on older participants’ performance. We replicated these results when we presented word and non-word stimuli integrated into a field of noise; older participants showed lower thresholds than younger participants and a similar early dropoff with lower noise contrasts. Consistent with these results, previous work by Bennett et al. (1999) examined differences in participants’ threshold noise contrast under a range of noise and positional uncertainly conditions, noting that older participants showed significant reductions in their thresholds. The similarity in results is interesting and non-obvious, since determining the orientation of a grating is a substantially different task than reading a word.

**Fig. 4:**
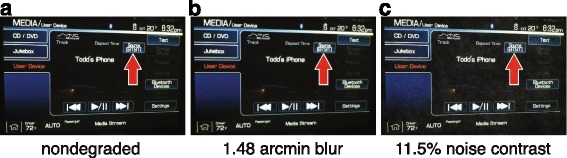
Visualization of the difference between younger and older participants’ performance in the degradation conditions. The *red arrow* in all three panels indicates a labeled button the driver might need to read at a glance in order to know where to tap on the screen. **a** Image of an in-vehicle interface (Ford Sync), without degradation. **b** Image from (**a**) with 1.48 arcmin blur applied, assuming a viewing distance of 70 cm, per the results of our experiments. **c** Image from (**a**) with 11.5% noise contrast added. Note that while the button remains relatively distinct, the text becomes somewhat harder to read with blur and noise, respectively

That older participants in our experiments had more difficulty with our task than their younger counterparts is not surprising (c.f. Adams & Hoffman, 1994; Mitzner & Rogers, 2003; Russell-Minda, Jutai, & Strong, 2007). However, the intriguing promise of our results is that by fitting our data to separate psychometric functions for older and younger participants, and then looking at the differences in fitted parameters, we can determine the level of blur or noise required to mimic the experience of an older participant for a younger participant. Based on our results in Experiment 1 (and corroborated by those in Experiment 2) and the primarily horizontal shift observed between the functions for older and younger participants, adding 1.48 arcmin of blur or 11.5% noise contrast will approximate the performance of an older participant (Fig. 4). These are relatively moderate degradations, but our results show that they significantly change legibility and that by approximating older participants’ performance, designs can be made more robust to age-related differences. Figure 4 provides a demonstration in which we use our results to visualize the differences between older and younger users to gain intuitions about how older users will perceive an in-vehicle interface. Figure 4b shows the original interface (shown in Fig. 4a) blurred with a Gaussian filter of standard deviation of 1.48 arcmin (assuming a viewing distance of 70 cm), as indicated by our results in Experiment 1. Figure 4c shows a similar manipulation with 11.5% added noise contrast, as specified by the shift in psychometric functions between older and younger adults in the noise condition of Experiment 1. Note that in our example, some text becomes difficult to read (the word “passenger” in the bottom left of the interface, the button labeled with “Source BTST” indicated by the arrow), to say nothing of the grayed-out buttons on the right hand side of the interface. While these degradations are not particularly large (1.48 arcmin of blur and 11.5% noise contrast), they do have an immediately noticeable impact on legibility of the interface components. Note that this visualization is not a substitute for actual user testing with a range of users as a product is developed, but it provides a useful early design tool to facilitate maximally legible and usable designs.

Critically, these results do not seem to be driven by underlying differences in performance on the lexical decision task, since our post-hoc tests found no differences in performance on non-degraded stimuli. Given that we observed no difference between older and younger participants in the no-degradation condition, we believe that the lexical decision task we used is easy enough for all our participants at a constant stimulus duration (250 ms) and that the differences we observed cannot be attributed to differential task difficulty on this core task. That being said, we did observe differences between older and younger participants in terms of reaction time, which is eminently worthwhile to consider in future designs. Even if older users can, if given sufficient time to do so, read a label in an interface (e.g. a button on the screen of their car’s navigation system), they may not have sufficient time to do so safely.

We do not claim to have exhausted the range of changes that occur in the aging visual system; far from it. Perhaps one of the most noticeable changes that occurs as we age is the reduced accommodation of the lens; an older lens will take longer to shift focus, and on the timescale that applies on the road, this delay may have its own deleterious effects on legibility. To some degree, this can be countered by changing text size for in-vehicle displays, but it is unlikely that this alone would solve the problem. Critically, our noise manipulation, which simulates the broadening of neuronal tuning with age, suggests that older users will have more difficulties extracting the information they need from what is presented. Building on our work here, we believe that there should also be some consideration of the fidelity with which text is rendered, because a variety of rendering assumptions, both in software and hardware (e.g. display resolution), with potential effects on legibility that may exacerbate the age-linked effects we have described. While we are not the first to consider how displays might be optimized for older users (see Kline (1994) for a discussion of potential optimizations and Adams and Hoffman (1994)), there have been profound changes in display technology since then, how designers intend them to be used and how users, in fact, use them, and our results speak to some of these newer user behaviors.

Our results have real-world implications for visual design and technology, particularly if the goal is to serve a wide range of potential users. Certainly, avoiding design decisions that blur text is an immediate implication, but this result also suggests that better displays (e.g. higher pixel density, resulting in higher fidelity images and text rendering) may avoid diminishing usability for older users. In addition, text can be perceptually degraded as an unavoidable consequence of moving through the world while trying to read the contents of a display; in a vehicle, the vibration of the vehicle can blur the display or a smartphone’s display can be harder to resolve because the user is attempting to read while walking. Our findings may suggest that considering all sources of degradation will improve usability, given the impact of blur and noise on legibility in our experiment. Simply put, just because reading a given button label seems easy now, does not mean it will be so—and the extra time that it takes to read a poorly designed label may not be time the driver can afford on the road.

## Conclusions

Our results provide critical information that will enable early-stage designs to be assessed for their relative legibility for older as well as younger users, much as simulations of colorblindness have been used to ensure usability for dichromats (Brettel, Viénot, & Mollon, 1997). By simulating the level of blur or noise that adversely impacts older users, a designer can gain an intuition about what makes text harder or easier for users to read at a glance, even if they would not intentionally blur or degrade the text in their design. Based on our work, we believe that for designs to be usable by the largest number of users, they should reflect the realities of the visual system, not only at the peak of our capacities, but also how it changes as people age.
